# Ecotoxicological effects of cypermethrin on indigenous climbing perch *(Anabas testudineus)*

**DOI:** 10.1016/j.heliyon.2024.e25723

**Published:** 2024-02-08

**Authors:** Sharmin Akter, Md. Abdullah-Al Mamun, Md. Sabbir Hossain, Arman Hossain, Md. Zobayer Rahman, Sarker Mohammed Ibrahim Khalil, Md. Moshiur Rahman, M.M. Mahbub Alam

**Affiliations:** Department of Fish Health Management, Sylhet Agricultural University, Sylhet-3100, Bangladesh

**Keywords:** Cypermethrin, *Anabas testudineus*, Histopathology, Hematology, LC_50_

## Abstract

Pesticides including cypermethrin (10% EC) are commonly used pesticide in tea gardens of Bangladesh possess distinct harmful effects on an aquatic community. The experiment was carried out to assess the ecotoxicological effects of cypermethrin (10%) concentrate on indigenous Climbing Perch (*Anabas testudineus*). A total of 120 *A*. *testudineus* (mean length 16 ± 2.67 cm and mean weight 31.6 ± 3.56 g) were exposed to the acute toxicity test when the lethal concentration 50 value (LC_50_) for 96 h was maintained at 1.00 ppm. Three different sub-lethal concentrations of 0.05 ppm (5%), 0.10 ppm (10%), and 0.20 ppm (20%) were used respectively as three treatments and a control of 0 ppm with three replicates each. Restlessness, erratic movement, increased opercular activities, loss of equilibrium, and irregular response to feeding were observed in all the treatments compared to control one. Concerning histopathological alterations, all the analyzed organs showed highest changes in the T3 (cypermethrin conc. 20%) compared to other treatments while T0 (0 ppm) had normal structure. The major changes in the gill were epithelial cell hyperplasia, necrosis, severe lamellar fusion and epithelial lifting; while necrotic proximal tubules, glomerular shrinkage, disrupted renal corpuscle of the kidney and nuclear pyknosis, degenerated hepatic cells and vacuolation were observed in the liver. Severe melanomacrophage centre (MMC), haemosiderosis and vacuolation were found in spleen. The effect of cypermethrin on the hematological parameters of experimental fish was also studied. Red blood cells, hemoglobin and hematocrit were decreased in the experimental groups and lowest value was in T3 while values of white blood cells were increased in the experimental groups compared to control one. Hence, the present observation revealed that pesticides even at low concentrations can cause harmful effects on *A. testudineus*.

## 1 Introduction

Toxic organic pollutants such as pesticides and other agrochemicals are frequently used in crop fields with many of them being non-biodegradable and poisonous [1]. Exposure to pesticide-contaminated water pushes fish and other aquatic biota at a substantially increased risk of death [2]. Pesticides can have a significant impact on different physiological and biochemical processes which ultimately cause significant harm to fish health [3]. This pesticide contamination of water can result in fish mortality which leads to the increment of the levels of unwanted chemicals in edible fish tissue or decreased production capacity of fish, which may damage the consumer health [4]. The persistence of many pesticides in the aquatic environment poses a serious hazard to the entire ecosystem, including human, and has caused bioaccumulation to become a growing problem for pesticides in recent years [5]. Pesticides are routinely employed in tea gardens yet their residues frequently end up in aquatic ecosystems. The reason behind choosing cypermethrin which is a synthetic pyrethroid, a broad-spectrum insecticide commonly used in agricultural fields to control a range of insect pests [6]. On the other hand, fish are completely reliant on water to breathe and contaminants are taken up by their food chain. These substances get into the fish's system and progressively modify the internal structure of the organs. These pesticides have an impact on fish tissues and they alter the histological and structural features of several organs [7]. To evaluate the ecotoxicological hazards, pesticide toxicity and effects on non-target aquatic creatures, including fish, are considered [8]. Stress-induced histopathologic alterations are excellent instruments for assessing the impact of xenobiotic toxicity [9]. Such as, determining the harmful impacts of pesticide pollutants on fish is crucial as pesticides directly damage key organs such as the gills, liver, heart, kidney, and intestine and they have a direct link to the food chain as well as pesticide contamination of water bodies [5]. Changes in the histology of tissues directly associated with pollutants such as the gills, liver, kidneys, and spleen, serve as important biomarkers for evaluating toxicity [10]. Now-a-days, the social media and other sorts of media has huge impact on the disease management through awaking the mass population and provide them valuable suggestions and information [[11], [12], [13]]. The use of social media has shown more promising during the COVID-19 time while most of country faced complete lock-down and mass population primarily relied on social media and other mass media [[14], [15], [16]].

The indigenous climbing perch (*A*. *testudineus*) is a wild freshwater teleost fish, was chosen as an experimental model for testing the acute toxicity of cypermethrin in this study because of its year-round availability and laboratory adaptability. It is popular as a protein rich and nutrient-dense food source and can survive for long periods of time without water [17]. The ability of fish to withstand extreme conditions could contribute to a rise in toxicant bioaccumulation [18]. In investigation of fish diseases, toxicological studies with environmental monitoring are increasingly looking into fish blood as an indicator of pathological and physiological alterations because there is a direct interaction between blood in the gill of fish and the water medium [9]. As a result, fish hematological tests could be used to determine fish health and water quality [19]. Fish behavioral changes serve as an effective index for measuring changes in environmental conditions and behavioral actions of fish may also a function as a defensive strategy [20].

Considering above mentioned facts, the objectives of the current study were to identify the behavioral responses, histopathological and hematological changes in *A. testudineus* after exposure to sub lethal quantities of commercial grade cypermethrin. This study is being undertaken on the persistence of *A. testudineus* against pesticides used in tea gardens. It is required to boost production volume along with customer safety. So, this study will be highly useful in determining pesticide residues on fish bodies as well as their survival rates at various pesticide dosage levels and potential damage to human health.

## 2 Materials and methods

### 2.1 Collection and rearing of experimental fishes

Juvenile *A. testudineus*, having mean length of 16 ± 2.67 cm and mean weight of 31.6 ± 3.56 g, were obtained from nearby natural water bodies including haor and beels at Jaintiapur Upazila, Sylhet. To observe the effect of cypermethrin, the experiment was performed in the Fish Disease Diagnosis and Pharmacology Laboratory under the Faculty of Fisheries, Sylhet Agricultural University. Potassium permanganate (KMnO_4_) solution (0.1%) was used to wash the collected *A. testudineus* which helped to prevent dermal contamination. The fishes were held in 4 glass aquaria (100 L capacity) for acclimatizing in the lab conditions before starting the experiment for 15 days [21,22]. About 40% water of the aquarium was changed on the daily basis to remove waste products and maintain water quality during acclimatization. Experimental fishes were kept under a 12-h light and dark photoperiod throughout the experiment and an aerator was employed to oxygenate the water continuously. Experimental fishes were fed with standard pellet feed (Osaka aquarium pellet feed, Thailand) at the rate of 2% body weight twice a day during acclimatization and toxicity test. Feeding was stopped 24 h prior to the beginning of the toxicity test experiment.

### Ethical statement

The sampling and animal handling was conducted following the criteria approved by the ethical committee of the Department of Fish Health Management, Sylhet Agricultural University, Bangladesh's (SAU/FHM/Ethical Committee/2020–01).

### 2.2 Water quality parameters

Water quality parameters were measured daily with YSI water quality multi-meter (USA) Total ammonia was measured by using of the ammonia test kit. During acclimation period, the average physico-chemical parameter of water was found as temperature 21.2 °C, dissolved oxygen 6.09 ppm, conductivity 138.6 (μS/cm), TDS 74.50 (mg/l), salinity 0.07, pH 7.28 and water pressure 760.6 (mmHg). All these parameters were calculated and analyzed after the whole experimental period for monitoring the effect of toxicity in the water.

### 2.3 Collection of pesticide and toxicity test

The importance of cypermethrin in agriculture and aquaculture led to its selection. It is a popular synthetic pyrethroid to control Argulus *(Argulus* sp.*)* diseases in aquaculture and to eradicate the cotton (*Gossipium borbedum*) pest [23]. During preparation for experiment, the required quantity of cypermethrin was taken directly from this 10% EC using a micropipette. The LC_50_ or the concentration that will kill 50% of a group of fish, have been determined in the majority of pesticide toxicology studies on fish. The fish are not fed during the test period and are normally exposed for 24, 48, or 96 h. Before starting the experiment, a trial experiment was conducted. For this, some fishes were collected for acute toxicity test. 60 fishes were divided into 6 treatments and were exposed to 0.25 ppm, 0.50 ppm, 0.75 ppm, 1 ppm, 1.25 ppm and 1.5 ppm. Finally, the LC_50_ value of cypermethrin for *A. testudineus* calculated as 1.0 ppm. The mortality rates were recorded and the dead fish were immediately removed from the tank. All tests were done at room temperature and the behavioral or external changes in fish body were observed during the exposure periods.

### 2.4 Experimental Setup

Four groups of triplicate glass aquaria were arranged with 10 fish in each aquarium and each glass aquarium was set up with aerator. From the reference of LC_50_ value, five times lower doses of were selected as sub-lethal concentrations for treatment groups. Throughout the experimental periods, fish from both the control group and cypermethrin treated groups were continuously monitored. Every day, uneaten feed was removed by siphoning and the siphoned water was then purified to put into the treatment tank (Table 1).

**Table 1:** Experimental design among different treatments.

Treatment	No. of fishes	Dosage (cypermethrin 10 EC)
Control (C)	10	Pesticide free water
Treatment 1 (T1)	10	5% of LC_50_
Treatment 2 (T2)	10	10% of LC_50_
Treatment 3 (T3)	10	20% of LC_50_

All treatment groups were fed with same commercial feed. The behavior of treated fish was monitored on a regular basis and compared to that of the control group to see if there was any divergence. At regular intervals eye movement, mucus secretion, swimming activity, fish descaling and body color change were observed throughout the experiment.

### 2.5 Blood collection for hematological studies

Three fish from each group were carefully chosen to collect blood at 15-days intervals as handling procedure was followed by the given rules and regulations of Animal Ethics Committees (AECs). Blood samples were collected from both control and treated fish, severing the caudal peduncle by a heparin coated syringe. Then preserved in 2 ml EDTA tubes containing fine layer of EDTA as anticoagulant. Hematological parameters were estimated according to Mythic 18 Blood Analyzer machine.

### 2.6 Histopathological studies

For histopathological investigations gill, liver, kidney and spleen samples of three *A. testudineus* from each treatment per replicate were collected at the end of exposure period. Small pieces of organs from controls and treated fish were fixed in 10% neutral buffered formalin for 72 h, washed with tap water. They were dehydrated through a graded series of ethanol. Then cleared in xylene, infiltrated in paraffin (70 °C) and sectioned at a thickness of 5 μm using rotary microtome machine. Sections were dried on a slide warmer at 37 °C for few hours, then stained routinely with haematoxyline and eosin as per the schedule. Then photographed and examined by light microscopy [24,25].

### 2.7 Statistical analysis

All of the information was coded and entered into a Microsoft Excel spreadsheet. Mean and Standard Error were used to express the data. At a 5% significance level, SPSS software (v20, Intel Inc.) was used to do a one-way analysis of variance (ANOVA) and the Duncan Multiple Range Test was employed to compare mean values. Differences were measured statistically significant (P < 0.05). Previously published articles have been followed to prepare high quality graphs [[26], [27], [28]].

## 3 Results

### 3.1 Mortality and behavioral changes in fish

**Fig. 1:**
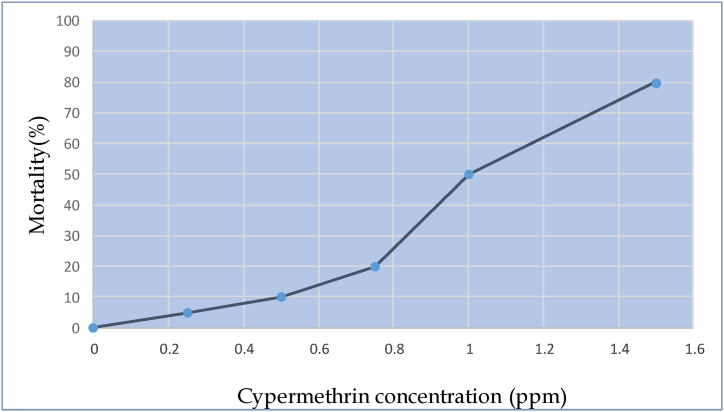
96 h LC_50_ of *A. testudineous* showing to different concentrations of cypermethrin.

In this experiment, control group showed no mortality with healthy status during the acclimatization. During the test exposure period, the cypermethrin-treated groups had a varied percentage of death in each dosing interval (Fig. 1). The 96 h LC_50_ value was found at 1 ppm but the lowest (5%) and highest (80%) mortality was observed at 0.25 ppm and 1.5 ppm, respectively in the total duration of the acute toxicity experiment. Exposure to 0.05, 0.10 and 0.20 ppm of cypermethrin caused substantial detectable behavioral changes in the test fishes. Fish demonstrate bodily irritation, suffocation immediately after treatment and they rush to the water's surface to gasp for air. After being exposed to various sub-lethal concentrations of cypermethrin, feed intake was lowered (Table 2).

**Table 2:** Behavioral changes in *A. testudineus* after sub-lethal exposure to cypermethrin.

Parameter	Treatment І	Treatment ІІ	Treatment ІІІ
Swimming activity	+++	+++	+++
Surfacing frequency	++	++	++
Opercular movements	++	++	+++
Mucus secretion	+	+	+
Body color	++	++	+++
Feeding activity	++	+	++

Abnormalities [(+) mild or quite regular, (++) moderate, (+++) severe].

### 3.2 Water quality parameters

Several water quality parameters were recorded in several times during the experiment to determine the fluctuation of different water quality parameters at different dosage of due to the presence of cypermethrin. However, water quality parameters did not differ with the various concentrations of pesticides including the control tank (Table 3).

**Table 3:** During the study period, following water quality parameters (Mean ± SE).

Water Quality Parameters	Treatment			
	Control	0.05 ppm	0.10 ppm	0.20 ppm
Temperature	25.2 ± 1	25.5 ± 1.3	25.35 ± 1.14	25.1 ± 1
pH	7.24 ± 0.065	7.415 ± 0.135	7.415 ± 0.16	7.215 ± 0.055
Water pressure	752.5 ± 0.15	752.7 ± 0.7	752.5 ± 0.5	752.45 ± 0.55
Dissolved oxygen	6.99 ± 0.61	6.845 ± 0.41	6.96 ± 0.1	7.11 ± 0.06
Conductivity	156 ± 28	157.5 ± 26.5	155.2 ± 23.2	153.45 ± 28.45
TDS	77 ± 13	76.5 ± 12.5	75.8 ± 10.8	79.5 ± 9.5
Salinity	0.07 ± 0.01	0.07 ± 0.01	0.07 ± 0.01	0.07 ± 0.01
Ammonia	0.375 ± 0.125	0.375 ± 0.125	0.375 ± 0.125	0.375 ± 0.125

### 3.3 Cypermethrin's effects on the histoarchitecture of gills

**Fig. 2:**
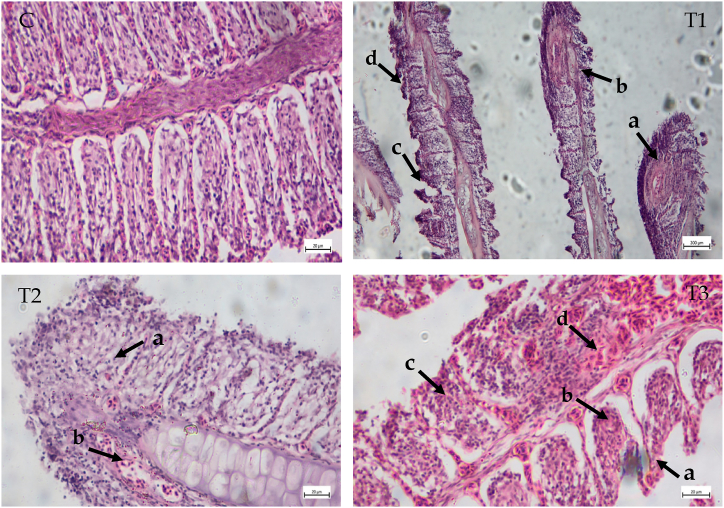
Histological studies of gill structure from control to treatment fish (C) Gill tissue of control fish (H and E × 40x); (T1) Gill tissue of *A. testudineus* exposed to 0.05 ppm (H and E × 10x): (a) desquamation and necrosis (b) degenerated gill lamellae (c) hyperplasia of epithelium (d) curling of secondary lamellae; (T2) Gill tissue of *A. testudineus* exposed to 0.10 ppm (H and E × 40x): (a) hyperplasia of epithelial cells and severe fusion of gill lamellae (b) hypertrophy (H); (T3) Gill tissue of A. testudineus exposed to 0.20 ppm (H and E × 40x): (a) epithelial lifting (b) hyperplasia of epithelial cell (c) fusion of adjacent lamella (d) hemorrhage. Scale Bar: 20 μm.

The gill arches of *A. testudineus* in the control group (fish that had not been exposed to cypermethrin) showed normal structure. Typical gill lesions (Fig. 2), Epithelial lifting, desquamation and necrosis, and epithelial cells hyperplasia were among the most common abnormalities discovered in the gills of indigenous *A. testudineus* after introduction with Cypermethrin (10% EC) (Table 4). Gill lamellar degeneration, Hyperplasia of epithelial cell, fusion and curling of secondary lamellae were prominent in treatment 1 group (Fig. 2T1). Severe fusion of gill lamellae was found in the treatment 2 in which fishes were exposed to 0.10 ppm (Fig. 2T2). In treatment 3 group, the severity of hyperplasia, hemorrhage and fusion increased as the toxicant concentration increased (Fig. 2T3).

**Table 4:** Histological alterations among several organs.

Organs	Anomalies	Control	Exposure to Cypermethrin (ppm)		
			0.05	0.10	0.20
Gills	Desquamation and necrosis	–	++	+	+
	Degeneration of gill lamellae	–	++	+	+
	Hyperplasia of epithelium	–	++	++	++
	Curling of secondary lamellae	–	+	+	+
	Fusion of gill lamellae	–	–	++	++
	Hypertrophy and Hemorrhage	–	+	+	+
	Epithelial lifting	–	+	–	++
Kidney	Glomerular shrinkage	–	++	+	++
	Melanomacrophage centre (MMC)	–	++	+	+++
	Vacuoles in tubules	–	+	+	+
	Pycnotic nuclei	–	+	++	+
	Increase in space between glomerulus and bowman's capsule.	–	+	+	+
	Inflammation	–	–	++	–
	Hypoplastic hematopoietic tissue	–	+	++	+
	Necrotic proximal tubules	–	+	++	+
	Disrupted renal corpuscle	–	+	++	+
	Tubular necrosis	–	–	+	++
Liver	Hemorrhages (H)	–	++	+	+
	Vacuolation of hepatocytes	–	+++	++	+
	Increase intracellular space.	–	+	+	+
	Nuclear pyknosis,	–	+	+	+
	Vacuolar degeneration,	–	+	++	+
	Congestion of central vein	–	–	++	++
	Necrosis	–	+	+	++
	Sinusoids	–	–	–	++
	Degenerated hepatic cell	–	+	+	++
Spleen	Melanomacrophage center (MMC)	–	++	++	+++
	Necrotic eosinophil	–	+	++	+
	Vacuolation	–	–	+	++
	Haemosiderosis	–	+	+	++

Level of histological alterations [(−) none, (+) mild, (++) moderate and acute (+++)].

### 3.4 Cypermethrin's effects on the histoarchitecture of kidney

**Fig. 3:**
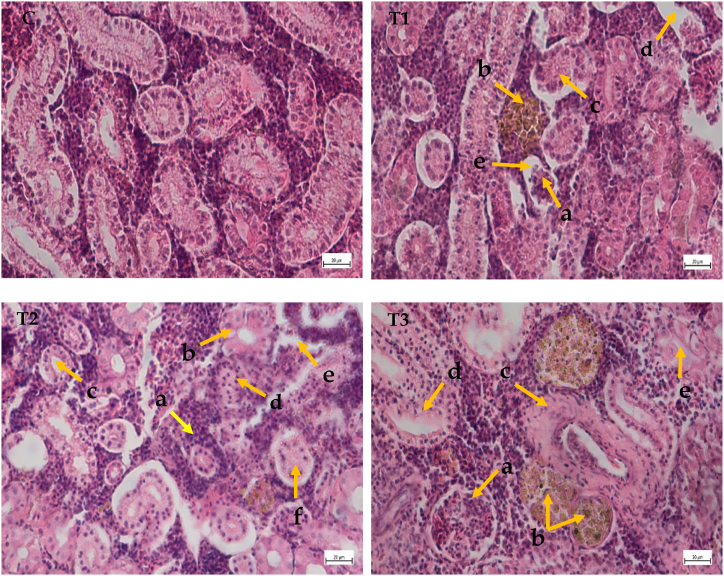
Histological studies of kidney structure from control to treatment fish (C) Kidney tissue of control fish (H and E × 40x); (T1) Kidney tissue of *A. testudineus* exposed to 0.05 ppm (H and E × 40x): (a) glomerular shrinkage (GS); (b) Pycnotic nuclei; (c) Melanomacrophage centre (MMC); (d) vacuoles; (e) Increase in space between glomerulus and bowman's capsule. (T2) Kidney tissue of *A. testudineus* exposed to 0.10 ppm (H and E × 40x): (a) inflammation; (b) hypoplastic hematopoietic tissue; (c) vacuoles in tubules; (d) necrotic proximal tubules; (e) enlarge intracellular space; (f) disrupted renal corpuscle. (T3) Kidney tissue of *A. testudineus* exposed to 0.20 ppm (H and E × 40x): (a) collapsing glomeruli (CG); (b) melanin and melanomacrophage centers; (c) ruptured kidney tubules; (d) degenerating renal tubule; (e) tubular necrosis. Scale Bar: 20 μm.

Kidney tubules, hematopoietic cells, blood vessels and renal corpuscles in the control group were found as normal and scientifically organized (Fig. 3C). Kidney tissue of *A. testudineus* under different treatments showed distinct histopathological changes (Table 3). In treatment 1, glomerular shrinkage (GS), Pycnotic nuclei, abnormalities in melanomacrophage centre and vacuoles were observed (Fig. 3T1). Inflammation, vacuoles in tubules, hypoplastic hematopoietic tissue (HHT), enlarged intracellular space, disrupted renal corpuscle and necrotic proximal tubules were found at the treatment 2 (Fig. 3T2). Ruptured kidney tubules (RKT), collapsing glomeruli, melanin and melanomacrophage centers, degenerated renal tubule and tubular necrosis were observed in the kidney tissue when fishes were exposed to 0.20 ppm under treatment 3 (Fig. 3T3).

### 3.5 Effect of cypermethrin on the histoarchitecture of liver

**Fig. 4:**
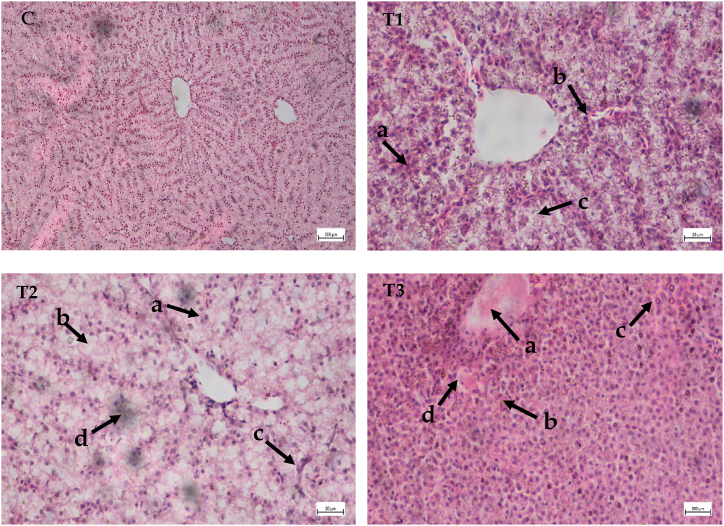
Histological studies of liver structure from control to treatment fish (C) Liver tissue of control fish (H and E × 10x); (T1) Liver tissue of *A. testudineus* exposed to 0.05 ppm (H and E × 40x): (a) hemorrhages (H), (b) severe diffuse vacuolation of hepatocytes (VH), (c) increase intracellular space. (T2) Liver tissue of *A. testudineus* exposed to 0.10 ppm (H and E × 40x): (a) nuclear pyknosis, (b) vacuolar degeneration, (c) congestion, (d) cloudy swelling. (T3) Liver tissue of *A. testudineus* exposed to 0.20 ppm (H and E × 40x): (a) congestion of central vein, (b) necrosis, (c) sinusoids, (d) degenerated hepatic cell. Scale Bar: 20 μm.

Hepatocytes, hepatopancreas in blood vessels and other liver cells were normal and grouped in a systematic manner in the control group (Fig. 4C). But cypermethrin exposed fish showed small or large vacuolation, necrosis, degeneration of hepatic cell, hemorrhage, increase in intracellular space, enlarged blood vessel in liver tissues (Table 4). Liver tissue of *A. testudineus* exposed to 0.05 ppm cypermethrin under the treatment 1 showed hemorrhages (H), severe diffuse vacuolation of hepatocytes (VH), increase intracellular space (Fig. 4T1). Treatment 2 in which fish exposed to 0.10 ppm, nuclear pyknosis, congestion, vacuolar degeneration and cloudy swelling was found (Fig. 4T2). Congestion of central vein, necrosis, sinusoids, degenerated hepatic cell (DHC) was found in the kidney tissue when fishes was exposed to 0.20 ppm under treatment 3 (Fig. 4T3).

### 3.6 Cypermethrin's effects on the histoarchitecture of spleen

**Fig. 5:**
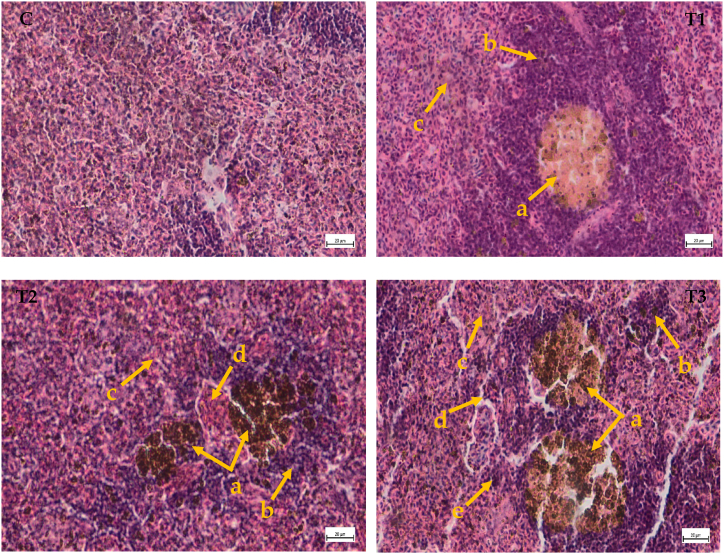
Histological studies of spleen structure from control to treatment fish (C) Spleen tissue of control fish (H and E × 10x); (T1) Spleen tissue of *A. testudineus* exposed to 0.05 ppm (H and E × 40x): (a) formation of MMC, (b) white pulp, (c) red pulp; (T2) Spleen tissue of *A. testudineus* exposed to 0.10 ppm (H and E × 40x): (a) MMC, (b) white pulp, (c) red pulp, (d) necrotic eosinophil; (T3) Spleen tissue of *A. testudineus* exposed to 0.20 ppm (H and E × 40x): (a) severe MMC (b) white pulp, (c) red pulp, (d) vacuolation, (e) Haemosiderosis. Scale Bar: 20 μm.

The current study discovered histological changes in the splenic sections of fish subjected to sublethal cypermethrin doses with a small number of lesions (Table 4). However, no deformities were found in spleen segments of control fish and the tissue structure was found as systematically arranged (Fig. 5C). Spleen tissue of *A. testudineus* exposed to 0.05 ppm cypermethrin under the treatment 1 indicated abnormally arranged white pulp and red pulp and formation of melano-macrophage centers (MMC) was also observed. In the spleen sections of fish which exposed to cypermethrin at dosage of 0.10 ppm and 0.20 ppm, several findings such as melanomacrophage centers (MMC), haemosiderosis, necrotic eosinophils and vacuolation were detected (Fig. 5T1-T3).

### 3.7 Studies on heamatological parameters

**Fig. 6:**
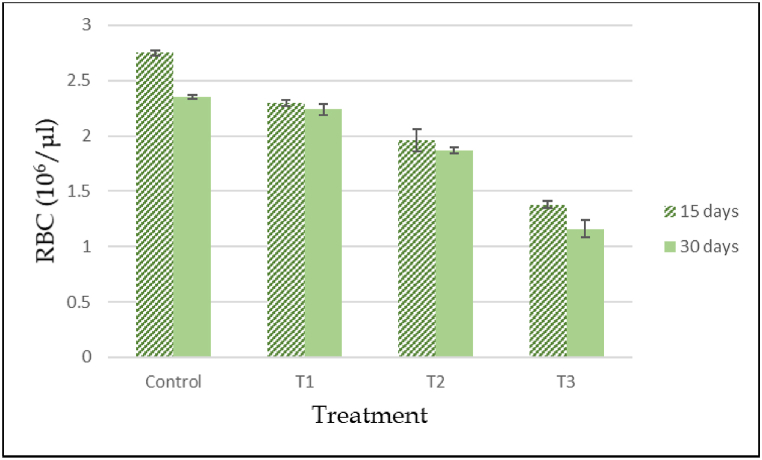
Total red blood cell count (RBC) in control and experimental fish exposed to ypermethrin's acute effect (10% EC). Data are expressed as mean ± SE.

**Fig. 7:**
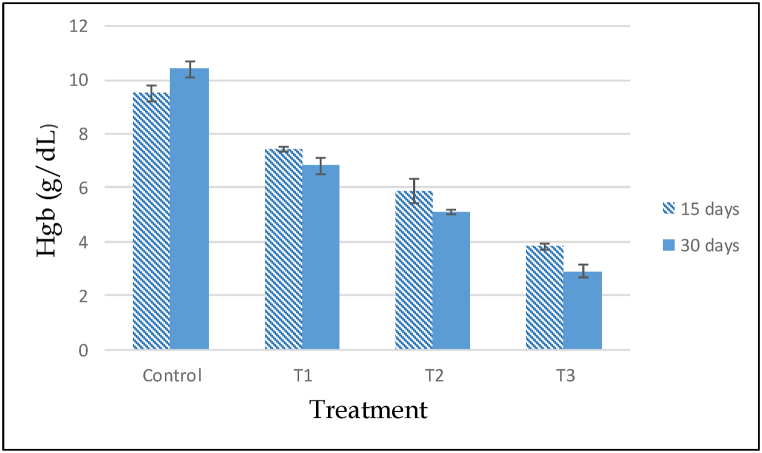
Hemoglobin count in control and experimental fish subjected to Cypermethrin's acute effect (10% EC). Data are expressed as mean ± SE.

**Fig. 8:**
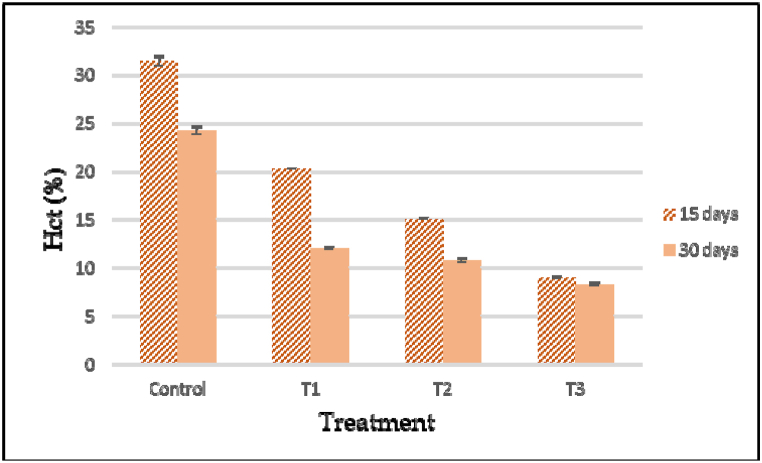
Hematocrit (Hct) of control and experimental fish exposed to acute effect of Cypermethrin (10%EC). Data are expressed as mean ± SE.

The effects of cypermethrin on the hematological parameters of *A. testudineus* subjected to different dosage of cypermethrin have been detected. According to the investigations, variations between selected parameters of control and treated fish were statistically significant. After 15 and 30 days of exposure, all fishes were exposed to cypermethrin showed a substantial change in RBC, WBC, Hb, Hct, MCH, MCV, MCHC and platelets compared to the control group and different doses of cypermethrin encouraged significant time and dose dependent (ANOVA, p < 0.05) have been summarized (Table 5). At different concentrations and time of exposure, the values of these variables were different (p < 0.05). When cypermethrin concentrations are below safety limit, RBC content is significantly reduced. After cypermethrin treatment, the total amount of RBC was found to be drastically reduced. The reduction was dose dependent; as the cypermethrin concentration increased, the RBC level decreased (Fig. 6). When compared to the control, the above values discovered a significant decrease (P < 0.05). Also, a significant reduction was recorded in hemoglobin (Hgb) and hematocrit (Hct) of cypermethrin treated group compared with the control fishes after 15 and 30 days of exposure (Fig. 7, Fig. 8). The Hematocrit (Hct) mean values were also gradually decreased with increased concentration of pesticide as related to the control group (Fig. 8).

**Table 5:** Hematological changes in *A. testudineus* as a function of sub-lethal cypermethrin (10% EC) concentrations during exposure treatment.

Hematological Parameters	Days of exposure	Concentration of Cypermethrin in ppm			
		0 ppm (Cont.)	0.05 ppm (T_1_)	0.10 ppm (T_2_)	0.20 ppm (T_3_)
WBCs (10^3^/μL)	15	97.47 ± 0.32^c^	111.4 ± 0.66^b^	111.97 ± 0.54^b^	129.6 ± 0.64^a^
	30	104.33 ± 0.41^d^	112.9 ± 0.32^c^	124.6 ± 0.50^b^	140.57 ± 0.62^a^
Lymphocytes (%)	15	95.23 ± 0.27^a^	90.5 ± 0.68^b^	88.133 ± 0.38^c^	88.7 ± 0.27^c^
	30	97.7 ± 0.21^a^	91.8 ± 0.321^c^	95.2 ± 0.46^b^	91.933 ± 0.29^c^
Monocytes (%)	15	2.87 ± 0.18^c^	5.23 ± 0.24^b^	6.034 ± 0.20^a^	5.8 ± 0.21^b^
	30	0.967 ± 0.18^c^	4.2333 ± 0.32^a^	3.233 ± 0.24^b^	4.2 ± 0.17^a^
Granulocytes (%)	15	1.9 ± 0.38^c^	4.27 ± 0.49^b^	5.833 ± 0.33^a^	5.5 ± 0.12^a^
	30	1.333 ± 0.20^b^	3.9667 ± 0.26^a^	1.567 ± 0.38^b^	3.867 ± 0.13^a^
RBCs (10^6^/μL)	15	2.75 ± 0.020^a^	2.3 ± 0.027^b^	1.96 ± 0.105^c^	1.38 ± 0.038^d^
	30	2.35 ± 0.02^a^	2.24 ± 0.05^a^	1.87 ± 0.03^b^	1.16 ± 0.08^c^
Hgb (g/dL)	15	9.5 ± 0.31^a^	7.433 ± 0.07^b^	5.88 ± 0.43^c^	3.82 ± 0.13^d^
	30	10.4 ± 0.29^a^	6.81 ± 0.31^b^	5.11 ± 0.10^c^	2.91 ± 0.25^d^
Hct (%)	15	31.47 ± 0.52^a^	20.29 ± 0.02^b^	15.08 ± 0.08^c^	9.02 ± 0.09^d^
	30	24.27 ± 0.41^a^	12.01 ± 0.11^b^	10.79 ± 0.23^c^	8.29 ± 0.17^d^
MCV (fL)	15	114.423 ± 1.04^a^	88.13 ± 0.97^b^	77.19 ± 3.98^c^	65.43 ± 1.83^d^
	30	103.4 ± 0.84^a^	53.8 ± 0.62^c^	57.89 ± .82^c^	71.92 ± 5.19^b^
MCH (pg)	15	34.04 ± .73^a^	33.56 ± 0.15^a^	29.93 ± 0.55^b^	27.72 ± 0.16^c^
	30	44.33 ± .84^a^	30.89 ± .78^b^	27.44 ± 0.16^c^	24.98 ± 0.48^d^
MCHC (g/dL)	15	29.85 ± 0.52^b^	37.99 ± 0.34^a^	39.02 ± 2.86^a^	42.44 ± 1.39^a^
	30	42.84 ± 0.48^b^	57.54 ± 2.09^a^	47.4 ± 1.82^b^	35.19 ± 3.25^c^
PLATELATES (cumm)	15	52.13 ± 0.24^d^	86.33 ± 3.03^c^	184.67 ± 1.45^b^	194.67 ± 2.40^a^
	30	296.33 ± 2.91^b^	218.33 ± 1.45^d^	252.67 ± 2.03^c^	591.67 ± 1.86^a^

*Note: Under each category, the mean values with different superscripts in rows differ considerably (P < 0.05). The data is expressed as mean ± SE.

**Fig. 9:**
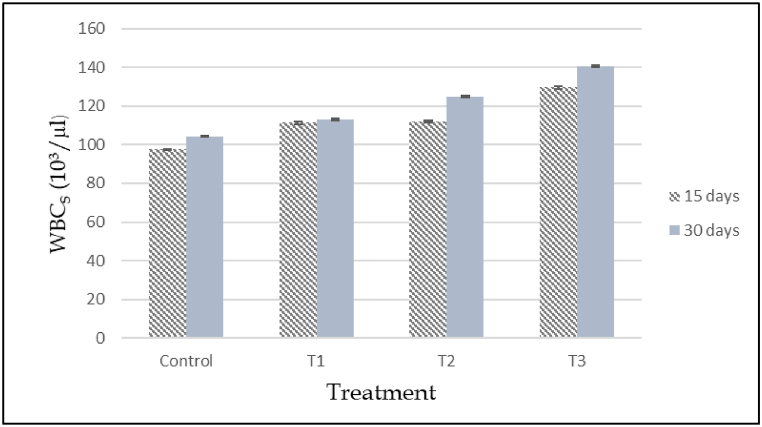
Total white blood cell count of control and experimental fish exposed to acute effect of Cypermethrin (10% EC). Data are expressed as mean ± SE.

On the other hand, WBC count was gradually increased from day 15 to day 30 (p < 0.05). Total WBC count was significantly increased in cypermethrin exposed group in where maximum in T3 group and minimum in control group (Fig. 9). In each treatment group, the numbers of lymphocytes and monocytes were described as statistically steady (Table 5). During the experimental periods, MCV and MCH showed a significant lowering tendency as cypermethrin toxicity increased, however MCHC showed no apparent changes (Table 5). The interactions of exposure length and concentration had no influence on the variables (p < 0.05).

## 4 Discussion

The 96-h LC_50_ of cypermethrin for *A. testudineus* was determined to be 1 ppm in the current investigation. In view of this, cypermethrin might be regarded as a moderately harmful substance to fish. Cypermethrin's acute toxicity depending on the fish species and region. However, significant time and concentration dependent abnormalities in tissue and blood parameters were observed when *A. testudineus* was subjected to sublethal dosages of cypermethrin. The physical and chemical characteristics of water influence pesticide toxicity, that's why, a physical and chemical study of the test water is necessary prior to starting an experiment. The resulted water quality of the tap water used in this study was within the normal range, indicating that the test water parameters were not the cause of fish mortality or other pathological and hematological disturbance.

### 4.1 Behavioral studies in ecotoxicology

Behavior is thought to be a useful tool in ecotoxicological and disease management study [[29], [30], [31]]. As a result, these findings were used as standards throughout the study. The control fish in this study acted naturally, they were active and coordinated in their motions. They were vigilant to the least disruption when feeding, but in the treatment, the fish exhibited unpredictable, irregular swimming patterns as well as a loss of equilibrium. Cypermethrin induced behavioral responses of the freshwater *Labeo rohita* were reported [32]. *Catla* shows loss of balance, abnormal swimming, frequently surfacing when exposed to methyl parathion at a dose range from 1 to 10 ppm [33]. When L. *rohita* exposed to acute toxic malathion, gulping air at the surface and swimming at the water surface were observed [34]. Previous studies reported a dose and duration dependent decrease in swimming activity in the treated groups; *Channa punctatus* tends to settle toward the bottom of the aquaria [20]. This could be related to endosulfan uptake by the gills and changes in gill structure, causing respiratory distress as well as an increased metabolic demand for oxygen or a disruption in metabolic reactions which caused energy depletion [20].

### 4.2 Histopathological studies of gill

In the present study, clear histopathological alternations were observed in the gill, liver, kidney and spleen of the fish exposed to cypermethrin. In toxicant impact assessment, histopathological observation is regarded a sensitive bio-monitoring approach for indicating the influence of toxicants on fish in pesticide-polluted aquatic habitats [35]. Gills are important organs in fish because they perform respiratory, osmoregulatory, and excretory activities that is extremely susceptible to toxic effects [35]. In this study necrosis, desquamation, lamellar epithelial lifting, hyperplasia of epithelial cells, hemorrhage and fusion of the secondary lamellae were observed in the gills after exposure to cypermethrin. However, epithelial enlargement reduces the respiratory surface area, lowering gas exchange efficiency and causing osmoregulatory dysfunction [36]. Gill epithelium and epithelial necrosis were direct responses to the deltamethrin action were reported [37]. Gill hyperplasia could be a protective mechanism, resulting in reduction of respiratory surface. Epithelial hyperplasia, curling of secondary lamellae, fusion of primary and secondary lamellae with increased concentration of malathion (10, 50, and 100 μg/L) in gills of *L*. *rohita* [38]. However, these deteriorated or severely damaged gills are unable to absorb dissolved oxygen from the water and transport it to the underlying capillaries through the membrane [39]. Several authors reported lamellar deformations on the gill lamellae as telangiectasis, hyperemia, and fusion of secondary lamellae in their study [40,41]. Edema with lamellar epithelium lifting and hyperplasia are common defense mechanisms that increase the pollutant–blood diffusion distance, impairing gaseous exchange [1].

### 4.3 Histopathological studies of kidney

In the kidney tissue, control fish consists of hepatopoetic cells, the full collecting duct and kidney tubule, an intact renal corpuscle with glomeruli and bowman's space, the distal and proximal tubule and an undamaged blood artery. The treatment of the fish was continued for 30 days with different dosages of cypermethrin (0.05, 0.10, and 0.20 ppm). hypoplastic hematopeotic tissue with ruptured kidney tubules, degraded renal tubules and increased intracellular space in the kidney, vacuoles, necrotic proximal tubules, hemorrhages and melanomacrophage centers were reported in this investigation. Similar histopathological alterations were observed in *Oreochromis niloticus* exposed to cypermethrin, including the manifestation of nucleus hypertrophy, tubular epithelial cell hypertrophy, and the presence of degenerated hyalin droplets [1]. Kidney of *Cirrhinus mrigala* subjected to monocrotophos, hypertrophied epithelial cells of renal tubules, narrowing of the glomerulus, pycnotic nuclei in tubular epithelium and enlargement of space inside the Bowman's capsule were observed [42] which is in agreement with our findings.

### 4.4 Histopathological studies of liver

For metabolism, xenobiotic purification, and hazardous chemical excretion, the liver is the most important organ [43]. The histopathological lesions in liver observed in the present study were vacuolar degeneration, nuclear pyknosis, congestion, narrowing of sinusoids, hypertrophy of hepatocytes, necrosis and hemorrhage. Hyperplasia, localized coagulative necrosis and hepatic mass disintegration were observed in *L*. *rohita* subjected to cypermethrin in previous investigations [1,23]. Increase intracellular space, cloudy swelling was also pragmatic in this study. Similar discoveries also have been reported in *Pangasianodon hypophthalmus* exposed to cypermethrin [39]. In another study on sumithion's effect on common carp (*C*. *carpio*) revealed disordered hepatopancreas and intensification of hepatopancreas lumen, expansion of lumen space and increment of intracellular space at different doses [44]. Different authors also detected different toxicological alterations in their liver tissues after exposing fish to various toxicants, such as *Glossogobius giuris* liver tissue sample treated with diazinon had reported deteriorating alterations [45] and *Mystus cavasius* for water pollution [46].

### 4.5 Histopathological studies of spleen

The spleen has been studied in many vertebrates as well as fish due to its importance in immunity-related processes which has been linked to lymphocyte depletion, white pulp proliferation, haemosiderosis, spleen enlargement and risen melanomacrophage centers in fish spleen due to environmental pollution [47]. Melanomacrophage centers (MMC) which is found in the spleen of fish as one of the most essential physiological features [48,49]. Stress causes a rise in the number of splenic MMCs, compatible with the findings of the current investigation, which identified a large number of MMCs in splenic sections of fish exposed to sub-lethal cypermethrin doses (10% EC). Haemosiderosis is a disease caused by the accumulation of haemosiderin in the body which is one the breakdown product of Hb, which could be the result of sublethal cypermethrin exposure in the fish *A. testudineus* [50]. Severity in the MMC as a fish spleen homeostatic mechanism to phagocytose increasing deposits of haemosiderin and other debris induced by tissue deterioration [51]. This is coherent with the findings of the current investigation, which revealed the presence of MMC in splenic sections of exposed fish that leads to vacuolation in European eel splenic tissue [52]. As continuation of the findings of previous studies, our investigation added some more data which will be useful in future research on fish immunotoxicology.

### 4.6 Heamatological studies

Blood is a pathophysiological reflection of the entire organism, so blood parameters are essential in determining the structural and functional condition of fish when exposed to toxicants. A decrease in hematological values in pesticide exposed fish indicated anemia which might be related to haemosynthesis, erythropoiesis and osmoregulatory malfunction or increase in the rate of erythrocyte destruction in haematopoetic organs [53]. On exposure to several pesticides, hematological measures (erythrocyte and leucocyte count, packed cell volume, hemoglobin content etc.) have been employed as bioindicators of toxicosis in fish [54]. The hematological response of *A. testudineus* treated to cypermethrin for 30 days was a substantial increase in RBC, Platelates and a significant decrease in RBC, Hb, HCT as well as a change in MCV, MCH, and MCHC count as compared to the control fish group. By long term exposure to cypermethrin, the failure or suppression of the fish's hematopoietic system that can be linked to the development or reduction in RBC, WBC and other hematological parameters in *A. testudineus*. The decline in RBC and haemoglobin, which resulted in haemolysis induced erythropenia and stress.

In our study, hemoglobin levels in the cypermethrin-treated group were considerably lower than the control group. A rapid decrease in haemoglobin content in *C*. *carpio* which response to toxicity and the authors speculated that this could be due to the generation of methaemoglobin and a direct response of O_2_ – radical [55]. However, after subchronic exposure of Diazinon to silver carp (*Hypophthalmichthys molitrix*), hematocrit content and Hb levels considerably increased [56] and the chronic levels of waterborne metals produces increased blood hemoglobin and hematocrit which causes disrupted gas exchange [57]. After long-term exposure to diazinon, decreased RBC, Hb and Hct levels have been documented in *Ctenopharyngodon idella* [58]. In our study, the WBC level of *A. testudineus* blood increased considerably during the cypermethrin exposure period. The rise in WBCs was a pathological response because WBCs stimulate the haemopoietic tissues and immune system during infestation including production of antibodies and chemical substances that work as defense against infection. After diazinon exposure, African catfish (*Clarias gariepinus*) had lower Hb, RBC, and WBC counts as well as higher MCV and MCH levels [4]. This type of leucocyte (WBC) response with the presence of pollutant and disease that induced tissue damage and significant nonspecific immune system abnormalities, resulting in increased WBC production [59,60].

Following the acute action of cypermethrin, MCV and MCH readings in the experimental group showed a considerable droplet (Table 4). In chlorpyrifos-exposed *O. niloticus*, significantly lower values of mean cell volume (MCV), mean cell hemoglobin (MCH), and mean corpuscular hemoglobin concentration (MCHC) indicate anemia [61]. Red blood cell enlargement or the discharge of red blood cells into the circulation, produced increased MCV and MCH during anxiety. The low MCHC content during acute therapy could be related to a decrease in Hb synthesis. The greater MCV and MCH values as well as the lower MCHC value indicated macrocytic anemia. Similar findings have been observed in teleost fish *C*. *carpio* after acute lindane exposure [62] as well as in freshwater fish *L*. *rohita* following chlorpyrifos exposure [19]. Thus, an increase in WBC in cypermethrin-exposed fish *A. testudineus* could be related to a stress-protective response and the large losses in RBC, haemoglobin, PCV, and MCV in our study could be attributable to haemolyses of RBC.

## 5 Conclusion

The study depicts deleterious effects of cypermethrin (10%) on the climbing perch (*A. testudineus*) which has been indiscriminately used in the tea garden of Bangladesh. To understand the effects, we have selected 4 important organs to see the histopathological changes and hematological alterations which are commonly considered as the best target organs in toxicological study. The observed changes in fish exposed to cypermethrin at different doses indicate the activation of mechanisms to counteract the toxic stress caused by this chemical. The use of cypermethrin (10% EC) can have detrimental impacts on fish organs, emphasizing the importance of avoiding its indiscriminate use in agriculture and aquaculture. Failure to address this issue could have adverse consequences on fish populations and production, ultimately posing a risk of endangered to extinction. These research findings will serve as valuable information for raising awareness among tea garden managers regarding the harmful effects of cypermethrin, and will facilitate the development of environmentally friendly agricultural pest management policies. Finally, further research is needed to fully understand the effects of cypermethrin on the aquatic animal health specially focusing on enzymatic and genomic level.

## Policy recommendation and implications

In our findings we have found significant damage on the health of climbing perch through analyzing histopathological and hematological parameters. We are worried of the indiscriminate use of this deleterious chemicals in the tea gardens of Bangladesh. In order to protect our aquatic diversity and health status, we need mass awareness of the harmful impact of cypermethrin which can be done through digital and social media. In addition, policy makers and implementation authority should come forward and act properly in order to restrict the import, production, sale and use of this deadliest chemical in the tea gardens and others crop culture system. Finally, government should ensure the implemtation of the “National Integrated Pest Management Policy (NIPMP)”- 2002 by the tea gardens owner/manager.

## Data availability

The data that support the findings of this study are available upon reasonable request from the corresponding author. Researchers interested in accessing the data can contact M. M. Mahbub Alam via email at mhbb_alam@sau.ac.bd.

## CRediT authorship contribution statement

**Sharmin Akter:** Writing – original draft, Investigation, Formal analysis, Data curation. **Md Abdullah-Al Mamun:** Writing – review & editing, Software, Methodology. **Md Sabbir Hossain:** Writing – original draft, Formal analysis, Data curation. **Arman Hossain:** Writing – original draft, Formal analysis, Data curation. **Md Zobayer Rahman:** Writing – original draft, Formal analysis, Data curation. **Sarker Mohammed Ibrahim Khalil:** Writing – review & editing, Visualization, Methodology. **Md Moshiur Rahman:** Writing – review & editing, Methodology, Formal analysis. **M.M. Mahbub Alam:** Writing – review & editing, Supervision, Project administration, Funding acquisition, Conceptualization.

## Declaration of competing interest

The authors declare that they have no known competing financial interests or personal relationships that could have appeared to influence the work reported in this paper.

## References

[1] Korkmaz N., Cengiz E.I., Unlu E., Uysal E., Yanar M. (2009). Cypermethrin induced histopathological and biochemical changes in Nile tilapia (*Oreochromis niloticus*), and the protective and recuperative effect of ascorbic acid. Environ Toxicol Pharmacol.

[2] Akter R., Pervin M.A., Jahan H., Rakhi S.F., Reza A.M., Hossain Z. (2020). Toxic effects of an organophosphate pesticide, envoy 50 SC on the histopathological, hematological, and brain acetylcholinesterase activities in stinging catfish (*Heteropneustes fossilis*). J Basic Appl Zool.

[3] Sabra F.S., Mehana E.S.E.D. (2015). Pesticides toxicity in fish with particular reference to insecticides. Asian J Agric Food Sci.

[4] Adedeji O.B., Adeyemo O.K., Agbede S.A. (2009). Effects of diazinon on blood parameters in the African catfish (Clarias gariepinus). Afr J Biotechnol.

[5] Islam M.S., Haque M.M., Uddin M.N., Hasanuzzaman M.D. (2019). Histopathology in the fish *Channa punctatus, Heteropneustes fossilis* and *Anabas testudineus* exposed to diazinon. Int J Fish Aquat Sci.

[6] Tiwari S., Tiwari R., Singh A. (2012). Impact of cypermethrin on fingerlings of common edible carp (*Labeo rohita*). Sci World J.

[7] Kumari R., Mishra B.K.P. (2020). Histoarchitectural alterations in Ovary of *Mystus tengara* exposed to Hybrid pesticide Chloropyriphos 50% + cypermethrin 5% EC. J Bio Eng Res.

[8] Moreira-Santos M., Ribeiro R., Araújo C.V. (2019). What if aquatic animals move away from pesticide-contaminated habitats before suffering adverse physiological effects? A critical review. Crit Rev Environ Sci Technol.

[9] Maurya P.K., Malik D.S., Yadav K.K., Gupta N., Kumar S. (2019). Haematological and histological changes in fish *Heteropneustes fossilis* exposed to pesticides from industrial waste water. Hum Ecol Risk Assess. (HERA).

[10] Bhuvaneshwari R., Padmanaban K., Babu Rajendran R. (2015). Histopathological alterations in muscle, liver and gill tissues of zebra fish *Danio rerio* due to environmentally relevant concentrations of organochlorine pesticides (OCPs) and heavy metals. Int J Environ Res.

[11] Azadi N.A., Ziapour A., Lebni J.Y., Irandoost S.F., Chaboksavar F. (2021). The effect of education based on health belief model on promoting preventive behaviors of hypertensive disease in staff of the Iran University of Medical Sciences. Archives of Public Health.

[12] Iorember P.T., Iormom B., Jato T.P., Abbas J. (2022). Understanding the bearable link between ecology and health outcomes: the criticality of human capital development and energy use. Heliyon.

[13] Abbas J., Wang D., Su Z., Ziapour A. (2021). The role of social media in the advent of COVID-19 pandemic: Crisis management, mental health challenges and Implications. Risk management and healthcare policy.

[14] Hafeez A., Dangel W.J., Ostroff S.M., Kiani A.G., Glenn S.D., Abbas J., Mokdad A.H. (2023). The state of health in Pakistan and its provinces and territories, 1990-2019: a systematic analysis for the Global Burden of Disease Study 2019. The Lancet Global Health.

[15] Yao J., Ziapour A., Toraji R., NeJhaddadgar N. (2022). Assessing puberty-related health needs among 10-15-year-old boys: a cross-sectional study approach. Archives de Pédiatrie.

[16] Geng J., Ul Haq S., Ye H., Shahbaz P., Abbas A., Cai Y. (2022). Survival in pandemic times: Managing energy efficiency, food diversity, and Sustainable Practices of nutrient intake amid COVID-19 Crisis. Frontiers in Environmental Science.

[17] Priyatha C.V., Chitra K.C. (2018). Acute toxicity of triclosan on the native freshwater fish, *Anabas testudineus* (Bloch, 1792): behavioral alterations and histopathological lesions. Int J Life Sci.

[18] Nordin I.L., Ibrahim N., Ahmad S.A., Hamidin N., Dahalan F.A., Shukor M.A. (2018).

[19] Ismail M., Ali R., Shahid M., Khan M.A., Zubair M., Ali T., Mahmood K.Q. (2018). Genotoxic and hematological effects of chlorpyrifos exposure on freshwater fish Labeo rohita. Drug Chem Toxicol.

[20] Harit G., Srivastava N. (2018). Behavioural alterations in *Channa punctatus* after exposure to endosulfan followed by subsequent recovery. Int J fish Aquat.

[21] Francis-Floyd R., Klinger R. (1997).

[22] Hoseini S.M., Tarkhani R. (2013). Effect of short‐term treatment with potassium permanganate on stress markers and blood biochemistry in goldfish *Carassius auratus*. Aquac Res.

[23] Sarkar B., Chatterjee A., Adhikari S., Ayyappan S. (2005). Carbofuran‐and cypermethrin‐ induced histopathological alterations in the liver of *Labeo rohita* (Hamilton) and its recovery. J Appl Ichthyol.

[24] Zaman M., Khalil S.M.I., Rahman M.Z., Hossain A., Al Mamun M.A., Rahman M.M., Alam M.M. (2023). Evaluation of histopathological alterations in the liver and kidney of Olive Barb (Puntius sarana, Hamilton 1822) as an indicator of the Surma River's pollution. Aquatic Sciences and Engineering.

[25] Rahman M.Z., Zaman M., Hossain A., Akter S., Rahman M.M., Mamun M.A.A., Alam M.M.M., Khalil S.M.I. (2024). Ovarian histology and histopathology of Olive Barb, Puntius sarana exposed to endocrine disrupting chemical (17-Α methyl Testosterone) in laboratory condition. Aquaculture Studies.

[26] Abbas J. (2021). Crisis management, transnational healthcare challenges and opportunities: the intersection of COVID-19 pandemic and global mental health. Research in Globalization.

[27] Schmidt C.A., Cromwell E.A., Hill E., Donkers K.M., Schipp M.F., Johnson K.B., Hay S.I. (2022). The prevalence of onchocerciasis in Africa and Yemen, 2000-2018: a geospatial analysis. BMC Med.

[28] Aqeel M., Rehna T., Shuja K.H. (2022). Comparison of Students' mental Wellbeing, Anxiety, Depression, and quality of Life during COVID-19's full and Partial (Smart) Lockdowns: a Follow-up study at a 5-Month interval. Front Psychiatry.

[29] Abbas J. (2020). The impact of Coronavirus (SARS-CoV2) Epidemic on Individuals mental health: the protective measures of Pakistan in managing and Sustaining Transmissible disease. Psychiatr Danub.

[30] Micah A.E., Bhangdia K., Cogswell I.E., Lasher D., Lidral-Porter B., Maddison E.R., Dieleman J.L. (2023). Global investments in pandemic preparedness and COVID-19: development assistance and domestic spending on health between 1990 and 2026. The Lancet Global Health.

[31] NeJhaddadgar N., Ziapour A., Zakkipour G., Abolfathi M., Shabani M. (2020). Effectiveness of telephone-based screening and triage during COVID-19 outbreak in the promoted primary healthcare system: a case study in Ardabil province, Iran. Z Gesundh Wiss.

[32] Marigoudar S.R., Ahmed R.N., David M. (2009). Cypermethrin induced respiratory and behavioural responses of the freshwater teleost, *Labeo rohita* (Hamilton). Vet Arh.

[33] Selvi R.T., Ilavazhahan M. (2015). Histopathological changes in gill tissue of the fish *Catla catla* exposed to sublethal concentration of pesticide methyl parathion and a heavy metal ferous sulphate. Biomed Pharmacol J.

[34] Shahbazi N.S., Mirvaghefi A., Gerami M.H., Ghafari F.H. (2015). Acute toxicity and behavioral changes of the gold fish (*Carassius auratus*) exposed to malathion and hinosan. Iran J Toxicol.

[35] Reiser S., Schroeder J.P., Wuertz S., Kloas W., Hanel R. (2010). Histological and physiological alterations in juvenile turbot (*Psetta maxima*, L.) exposed to sublethal concentrations of ozone-produced oxidants in ozonated seawater. Aquac.

[36] Sakuragui M.M., Sanches J.R., Fernandes M.N. (2003). Gill chloride cell proliferation and respiratory responses to hypoxia of the neotropical erythrinid fish *Hoplias malabaricus*. J Com Physiol.

[37] Cengiz E.I. (2006). Gill and kidney histopathology in the freshwater fish *Cyprinus carpio* after acute exposure to deltamethrin. Environ Toxicol Pharmacol.

[38] Karmakar S., Patra K., Jana S., Mandal D.P., Bhattacharjee S. (2016). Exposure to environmentally relevant concentrations of malathion induces significant cellular, biochemical and histological alterations in *Labeo rohita*. Pestic Biochem Phys.

[39] Monir M.S., Doulah M.A.U., Rahman M.K., Akhter J.N., Hossain M.R. (2015). Effect of cypermethrin on the histoarchitecture of gills and liver of a freshwater catfish. Pangasianodon hypophthalmus. Asian J Med Biol Res.

[40] Benli A.C.K., Özkul A. (2010). Acute toxicity and histopathological effects of sublethal fenitrothion on Nile tilapia, *Oreochromis niloticus*. Pestic Biochem Physiol.

[41] Mazumder S.K., Debi S., Das S.K., Salam M.A., Alam M.S., Rahman M.L., Mamun M.A.A., Ibrahim Khalil S.M., Pandit D. (2024). Effects of extreme-Ambient temperatures in silver barb (*Barbonymus gonionotus*): metabolic, hemato-biochemical responses, enzymatic activity and gill histomorphology. Water.

[42] Velmurugan B., Selvanayagam M., Cengiz E.I., Unlu E. (2007). The effects of monocrotophos to different tissues of freshwater fish *Cirrhinus mrigala*. Bull Envirn Contam Toxicol.

[43] Wolf J.C., Wheeler J.R. (2018). A critical review of histopathological findings associated with endocrine and non-endocrine hepatic toxicity in fish models. Aquat Toxicol.

[44] Shira M., Chowdhury P., Rahman M.S., Haque S.M., Shahjahan M. (2020). Effects of organophosphate insecticide, sumithion on histopathology of common carp (*Cyprinus carpio*) in the natural pond condition. Int J Agric Res Innov Technol.

[45] Banik U., Rahman M.M., Khanam T., Mollah M.F.A. (2016). Histopathological changes in the gonads, liver, and kidney of *Glossogobius giuris* exposed to sub-lethal concentration of diazinon. Progress Agric.

[46] Karim M.A., Rohani M.F., Hasan A.M., Farhad F.B., Alam M.M., Khalil S.M.I., Islam S.M. (2022). Health status monitoring of *Mystus cavasius* through histological aberrations of liver and kidney due to the deterioration of water Physico-chemical parameters in Surma River. Environmental Chemistry and Ecotoxicology.

[47] Sayed R.K.A., Zaccone G., Capillo G., Albano M., Mokhtar D.M. (2022 May 20). Structural and functional aspects of the spleen in molly fish *Poecilia sphenops* (valenciennes, 1846): synergistic interactions of stem cells, neurons, and immune cells. Biology (Basel).

[48] Agius C., Roberts R.J. (2003). Melano‐macrophage centres and their role in fish pathology. J Fish Dis.

[49] Khalil S.M.I., Orioles M., Tomé P., Galeotti M., Volpatti D. (2024). Current knowledge of lactococcosis in rainbow trout: pathogenesis, immune response and prevention tools. Aquaculture.

[50] Hibiya T. (1982).

[51] Kaleeswaran B., Ilavenil S., Ravikumar S. (2012). Changes in biochemical, histological and specific immune parameters in *Catla catla* (Ham.) by Cynodon dactylon (L.). J King Saud Univ Sci.

[52] Spazier E., Storch V., Braunbeck T. (1992). Cytopathology of spleen in eel *Anguilla anguilla* exposed to a chemical spill in the Rhine River. Dis Aquat Org.

[53] Jenkins F., Smith J., Rajanna B., Shameem U., Umadevi K., Sandhya V., Madhavi R. (2003). Effect of sub-lethal concentrations of endosulfan on hematological and serum biochemical parameters in the carp *Cyprinus carpio*. Bull Environ Contam Toxicol.

[54] Singh N.N., Srivastava A.K. (2010). Haematological parameters as bioindicators of insecticide exposure in teleosts. Ecotoxicol.

[55] Ramesh M., Saravanan M. (2008). Haematological and biochemical responses in a freshwater fish *Cyprinus carpio* exposed to chlorpyrifos. Int J Integr Biol.

[56] Hedayati A., Niazie E.H.N. (2015). Hematological changes of silver carp (*Hypophthalmichthys molitrix*) in response to Diazinon pesticide. J Environ Health Sci Eng.

[57] Chowdhury M.J., McDonald D.G., Wood C.M. (2004). Gastrointestinal uptake and fate of cadmium in rainbow trout acclimated to sublethal dietary cadmium. Aquat. Toxicol..

[58] Pourgholam R., Soltani M., Hassan M.D., Ghoroghi A., Nahavandi R., Pourgholam H. (2006). Determination of diazinon LC50 in grass carp (*Cetenopharyngodon idella*) and the effect of sublethal concentration of toxin on some hematological and biochemical indices. Iran J Fish Sci.

[59] Das B.K., Mukherjee S.C. (2003). Toxicity of cypermethrin in *Labeo rohita* fingerlings: biochemical, enzymatic and haematological consequences. Comp. Biochem. Physiol. Part - C: Toxicol. Pharmacol..

[60] Khalil S.M.I., Bulfon C., Galeotti M., Acutis P.L., Altinok I., Kotzamanidis C., Vela A.I., Fariano L., Prearo M., Colussi S., Volpatti D. (2023). Immune profiling of rainbow trout (*Oncorhynchus mykiss*) exposed to *Lactococcus garvieae*: evidence in asymptomatic versus symptomatic or vaccinated fish. Journal of Fish Diseases.

[61] Samajdar I., Mandal D.K. (2015). Acute toxicity and impact of an organophosphate pesticide, chlorpyrifos on some haematological parameters of an Indian minor carp, *Labeo bata* (Hamilton 1822). Int J Environ Sci.

[62] Saravanan M., Kumar K.P., Ramesh M. (2011). Haematological and biochemical responses of freshwater teleost fish *Cyprinus carpio* (Actinopterygii: cypriniformes) during acute and chronic sublethal exposure to lindane. Pestic Biochem Phys.

